# One-Pot Synthesis for Doped Amorphous Carbon-Based Compounds: Influence of ZnO Dopant on the Charge Transfer Efficiency

**DOI:** 10.3390/nano15191486

**Published:** 2025-09-29

**Authors:** Bernardo Alberto Vargas-Vidal, Esperanza Baños-López, María del Rosario Munguía-Fuentes, Yazmín Mariela Hernández-Rodríguez, Oscar Eduardo Cigarroa-Mayorga

**Affiliations:** Department Advanced Technologies, UPIITA-Instituto Politécnico Nacional, Av. IPN 2580, Ciudad de México 07340, Mexico; bvargasv1600@alumno.ipn.mx (B.A.V.-V.); esperanza_banoslo@hotmail.com (E.B.-L.); mrmunguiaf@ipn.mx (M.d.R.M.-F.)

**Keywords:** ZnO doping, hydrothermal synthesis, photocatalysis, rhodamine 6G degradation, charge transfer enhancement

## Abstract

Amorphous carbon (a-C) materials have attracted significant attention for environmental remediation due to their chemical stability and high surface area; however, their photocatalytic activity remains limited by rapid electron–hole recombination. In this study, ZnO-doped amorphous carbon (a-C@ZnO) composites were synthesized via a one-pot hydrothermal method to enhance charge separation and photocatalytic performance. The synthesis involved the carbonization of glucose and the incorporation of zinc species under controlled conditions, resulting in composites with varying ZnO contents. The physical and chemical properties of the materials were thoroughly characterized by SEM, Raman spectroscopy, and X-ray photoelectron spectroscopy, confirming the successful integration of ZnO within the carbon matrix and the formation of Zn–O–C chemical bonds. Photocatalytic tests, evaluated through the degradation of rhodamine 6G under UV irradiation, demonstrated that ZnO doping significantly improved photocatalytic efficiency, with the a-C@ZnO_0.75_ sample achieving a 72% degradation rate and the highest kinetic rate constant. The enhancement was attributed to improved charge transfer and reactive oxygen species generation facilitated by the ZnO–a-C interface. These findings highlight the potential of ZnO-doped amorphous carbon composites as effective, low-cost photocatalysts for water purification applications.

## 1. Introduction

Charge transfer phenomena are fundamental to a vast array of physical, chemical, and biological processes that sustain modern technological society [1]. From solar energy harvesting to environmental remediation, the ability to control the movement of electrons across interfaces directly influences the efficiency, stability, and functionality of a wide variety of systems [2]. In a global context increasingly defined by energy scarcity, environmental degradation, and technological advancement, understanding and optimizing charge transfer mechanisms is crucial for developing sustainable solutions [3,4]. Enhanced charge transfer processes underpin advances in photocatalysis, energy storage, optoelectronics, and sensor technology and remain at the forefront of materials science research [5,6,7]. At its core, charge transfer refers to the movement of electrons or holes between different regions, molecules, or phases in a system, often facilitated by an external stimulus such as light, heat, or an electric field [8]. In energy conversion applications, particularly photocatalysis, and photovoltaics, efficient charge transfer is critical for minimizing recombination losses and maximizing the generation of reactive species or electric currents [9,10]. Similarly, in electrochemical energy storage devices such as batteries and supercapacitors, charge transfer rates dictate the storage capacity, charging speed, and cycle life of the devices [11,12]. Even in biological systems, such as photosynthesis and respiration, precise charge transfer reactions drive the fundamental biochemical transformations of life [13]. The omnipresence of charge transfer processes in both artificial and natural systems underscores its importance across disciplines and applications. The practical relevance of charge transfer spans multiple technological domains. In photocatalysis, efficient charge separation and transfer enable the degradation of persistent organic pollutants, offering environmentally friendly methods for water purification and air cleaning [14]. In solar energy conversion, photovoltaic devices rely on rapid and directional charge transfer at interfaces to generate electric current from sunlight [15]. In electronics and optoelectronics, charge transfer governs device behavior, from organic light-emitting diodes to field-effect transistors [16]. Emerging technologies such as artificial photosynthesis, next-generation sensors, and quantum information processing also critically depend on the control of interfacial and intramolecular charge transfer pathways [17]. Thus, improving the understanding and engineering of charge transfer mechanisms is pivotal to addressing some of the most pressing scientific and societal challenges. Among the materials widely investigated for facilitating and optimizing charge transfer, carbon-based nanostructures have attracted significant interest due to their unique combination of chemical, mechanical, and electronic properties [18]. Materials such as graphene, carbon nanotubes, amorphous carbon, and graphitic carbon nitride possess high electrical conductivity, excellent chemical stability, and large surface areas, making them ideal platforms for charge storage and transfer [19]. Particularly, amorphous carbon (a-C), with its tunable sp^2^/sp^3^ hybridization and defect-rich structure, offers versatile opportunities for tailoring charge transport properties [20]. The inherent structural disorder of a-C provides abundant active sites for electron interaction but also presents challenges by introducing localized states that can trap carriers and limit mobility [21]. Consequently, strategies to enhance charge transfer in amorphous carbon-based materials are essential for fully realizing their potential across applications. In addition, one of the most effective approaches to improve charge transfer in nanometric systems is the formation of heterostructures or hybrid composites [22]. By coupling carbon materials with other functional elements such as metals, metal oxides, or semiconductors, it is possible to engineer charge separation pathways, create internal electric fields, and extend light absorption ranges [23]. In addition, heterostructures provide complementary properties that neither component could achieve alone: while the carbon matrix offers conductivity and surface reactivity, the secondary phase can introduce new electronic states or catalytic functionalities. Among different materials, the properties of ZnO [14,23] made it an important candidate to explore a-C-based complex structures for enhancement of charge transfer.

In this context, the present research contributes to the broader understanding of charge transfer enhancement in carbon-based materials by proposing a simple accessible method for incorporating ZnO into amorphous carbon via one-pot hydrothermal synthesis. The effect of ZnO on the charge transfer behavior is discussed.

## 2. Materials and Methods

### 2.1. Materials and Reagents

All chemical reagents used in this study were of analytical grade and employed without further purification. The carbon precursor utilized for the synthesis of amorphous carbon (a-C) was D-(+)-glucose anhydrous (≥99.5%, Sigma-Aldrich, St. Louis, MO, USA, SKU: 1181302). Zinc chloride hexahydrate (ZnCl_2_·6H_2_O, Sigma-Aldrich, SKU: 429430) served as the zinc source for doping purposes, selected due to its high solubility and reactivity in aqueous media. Rhodamine 6G (R6G, Sigma-Aldrich, SKU: 56226) was employed as the model organic dye for evaluating the photocatalytic activity of the synthesized samples as an indirect way to measure the charge transfer [14]. Deionized water (resistivity ≥18.2 MΩ·cm) was used throughout all synthesis, washing, and dilution procedures to avoid contamination and ensure reproducibility. All solutions were prepared freshly before synthesis or experimentation using calibrated glassware. Storage and handling of materials were conducted under ambient laboratory conditions.

### 2.2. Synthesis of Amorphous Carbon

Amorphous carbon (a-C) was synthesized via a hydrothermal method using glucose as the carbon precursor. Thus, 10 mL of an aqueous glucose solution with a 0.5 M concentration was prepared by dissolving anhydrous D-(+)-glucose in deionized water. The solution was stirred at room temperature until complete dissolution was achieved. The prepared solution was then transferred into a 50 mL Teflon-lined stainless-steel autoclave, ensuring the vessel was sealed tightly to maintain pressure during the reaction. The autoclave was placed in a laboratory oven and heated at 200 °C for 4 h. This temperature and reaction time were selected based on preliminary trials to ensure optimal carbonization while avoiding crystallization. Upon completion of the reaction, the autoclave was allowed to cool naturally to room temperature. The resulting product appeared as a dark-brown to black solid, indicating successful carbonization. The product was collected by centrifugation at 6000 rpm for 10 min, washed three times with deionized water to remove unreacted precursors, and then dried at 60 °C for 12 h in a vacuum oven. To explore the influence of precursor concentration and reaction time on the yield and quality of a-C, multiple synthesis conditions were evaluated by varying glucose molarity and reaction duration, while maintaining the hydrothermal temperature constant at 200 °C. All synthesized samples were stored in airtight containers for subsequent characterization and photocatalytic evaluation.

### 2.3. Synthesis of ZnO-Impurified Amorphous Carbon

ZnO-doped amorphous carbon (a-C@ZnO) was synthesized using a one-pot hydrothermal method, designed to enable simultaneous carbonization and zinc incorporation in a single step. The procedure involved preparing two aqueous solutions: one containing anhydrous D-(+)-glucose as the carbon precursor and the other containing zinc chloride hexahydrate as the zinc source. The two solutions were mixed in stoichiometric ratios to yield final zinc weight percentages of 0.25%, 0.5%, 0.75%, and 1.0% in the solid product. The resulting homogeneous solution was transferred into a 50 mL Teflon-lined stainless-steel autoclave, which was sealed and placed in an oven at 200 °C for 4 h to initiate the hydrothermal reaction. After cooling to room temperature, the solid products were recovered by centrifugation at 6000 rpm for 10 min, followed by triple washing with deionized water to eliminate residual ions and unreacted species. The washed samples were then dried at 60 °C for 12 h in a vacuum oven. All synthesized composites were labeled according to their ZnO nominal weight percentage as a-C@ZnO_0.25_, a-C@ZnO_0.50_, a-C@ZnO_0.75_, and a-C@ZnO_1.0_, with a-C@ZnO_0.0_ representing the undoped reference sample.

### 2.4. Physical and Chemical Characterization

The surface morphology and particle agglomeration of all samples were evaluated using a JEOL JSM-7401F field emission scanning electron microscope (JEOL, Tokyo, Japan). The analysis was performed on gold-coated powder samples to prevent charging effects during imaging. The internal structure and crystallinity of the ZnO-impurified a-C samples were analyzed using a JEOL JEM-2100F transmission electron microscope operated at 200 kV. Powdered samples were ultrasonically dispersed in ethanol and drop-cast onto carbon-coated copper grids. High-resolution TEM images were acquired to evaluate nanoparticle dispersion. The degree of disorder and graphitic structure within the carbon matrix was studied using a LabRAM HR800 Raman spectrometer (Horiba Jobin Yvon, Edison, NJ, USA) equipped with a 633 nm He–Ne excitation laser. Spectra were recorded in the range of 1000–3200 cm^−1^. The intensity ratio between the D band (~1350 cm^−1^) and G band (~1580 cm^−1^) was used to estimate the defect density and structural modifications induced by ZnO doping [23]. On the other hand, the elemental composition and chemical bonding states were analyzed using a Thermo Scientific K-Alpha XPS system (Thermo Fisher Scientific, Waltham, MA, USA) equipped with a monochromatic Al Kα source (hν = 1486.6 eV). Survey and high-resolution spectra were acquired for the C 1s, O 1s, and Zn 2p regions.

### 2.5. Charge Transfer Evaluation

The charge transfer activity of the synthesized amorphous carbon (a-C) and ZnO-doped amorphous carbon (a-C@ZnO) samples was assessed through the degradation of rhodamine 6G under ultraviolet (UV) irradiation as an indirect measurement of charge carriers’ behavior [14]. A stock solution of 0.1 M R6G was prepared using deionized water and then serially diluted to obtain a working concentration of 3.125 × 10^−3^ M, optimized for accurate spectrophotometric detection. In a typical photocatalytic test, 2.0 mL of the R6G working solution was placed into a polystyrene UV cuvette (10 × 10 × 45 mm, SARSTEDT, Nümbrecht, Germany), and 5 mg of the photocatalyst powder was added. The suspension was irradiated using a UV lamp (λ = 365 nm) under ambient temperature and pressure, without stirring. UV–Vis absorbance spectra were recorded at regular intervals (every 30 min for 150 min total) using a Hanon i3 UV–visible spectrophotometer (Wuhan, China) to monitor the decrease in R6G concentration. The degradation efficiency was calculated using Equation (1).

(1) $$Degradation\;efficiency\;\left(\%\right)=\left(1-\frac{C_{t}}{C_{0}}\right)\times 100$$

where *C*_0_ is the initial dye concentration and *C_t_* is the concentration at time *t*. The apparent reaction kinetics were determined using a first-order kinetic model as shown in Equation (2).

(2) $$ln\left(\frac{C_{0}}{C_{t}}\right)=kt$$

where *k* is the rate constant (min^−1^) and *t* is the irradiation time. All measurements were performed in triplicate to ensure reproducibility and average values were reported. The photocatalytic performance was compared across all samples to evaluate the effect of ZnO doping concentration on dye degradation efficiency and charge transfer behavior.

## 3. Results and Discussion

### 3.1. Carbon Synthesis Based on Glucose

**Figure 1:**
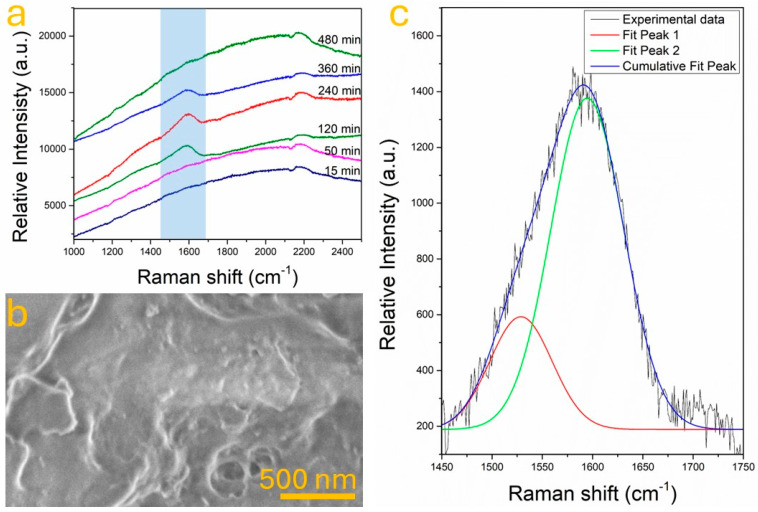
(**a**) Raman spectra comparison of samples synthesized at different durations under the hydrothermal process. (**b**) FESEM image of a-C obtained at 200 °C for 240 min. (**c**) Deconvoluted Raman spectrum of the sample shown in (**b**), in the range of 1450 cm^−1^ to 1750 cm^−1^.

Figure 1a shows the Raman spectra of samples synthesized by hydrothermal method at different times (15 min, 50 min, 120 min, 240 min, 360 min, and 480 min) at a constant temperature of 200 °C. All samples exhibit broad Raman features characteristic of disordered carbon, notably the D band (~1350 cm^−1^) associated with defects and disordered sp^2^ domains [24] and the G band (~1580 cm^−1^) attributed to the stretching vibration of sp^2^ carbon atoms in graphitic networks [25]. As synthesis time increases, both bands become more pronounced, indicating progressive carbonization and structural ordering of the carbonaceous matrix. At short reaction times (15–50 min), the Raman intensity is relatively low, and the bands appear broadened and less defined, suggesting that the carbon phase is underdeveloped, with incomplete dehydration and polymerization of glucose-derived intermediates. As the time increases to 120 and 240 min, the D and G bands become distinguishable, with intensities increasing significantly, denoting the formation of a denser and more ordered carbon framework. On the other hand, Figure 1c shows the deconvoluted Raman spectrum for the sample synthesized at 240 min. The fitting reveals two overlapping peaks corresponding to the D band (~1510 cm^−1^) and G band (~1590 cm^−1^). The intensity ratio I_D_/I_G_ for this sample is calculated to be approximately 1.23, indicating a high degree of structural disorder with a dominant presence of edge-plane defects and amorphous regions [26,27]. For comparison, the sample synthesized at 120 min showed an I_D_/I_G_ ratio of ~1.08, while the 360 and 480 min samples reached values of 1.26 and 1.29, respectively.

These values are in agreement with previous literature on amorphous carbon produced by hydrothermal or low-temperature pyrolytic methods. Typical I_D_/I_G_ ratios for a-C synthesized under mild conditions (≤250 °C) range from 1.0 to 1.5, depending on the precursor, reaction conditions, and degree of graphitization [28]. An I_D_/I_G_ ratio around 1.2–1.3 is often reported for partially disordered, sp^2^-rich amorphous carbon suitable for functionalization and hybrid material formation [29]. In addition, the FESEM image in Figure 1b further confirms the morphological characteristics of the sample synthesized at 240 min. The surface displays a compact carbonaceous layer with a wrinkled and irregular texture. This morphology is consistent with that of polymer-derived amorphous carbon obtained under hydrothermal conditions and aligns with the Raman-determined structural disorder [30]. The progressive increase in I_D_/I_G_ with time could indicate the evolution of structural defects and clustering of aromatic domains [31]. While longer reaction times promote further carbonization and crosslinking, they also introduce more topological defects and sp^3^-type distortions, contributing to the rise in disorder signatures. This balance between sp^2^ domain formation and defect density is critical in determining the electronic properties of a-C materials, particularly for applications where surface reactivity and electron transfer efficiency are essential [32,33]. In this study, the 240 min synthesis condition was identified as an optimal compromise between structural development, defect richness, and practical yield. The material obtained under these conditions possesses sufficient sp^2^ hybridization to support electronic conduction while maintaining a high density of active sites for further interaction with dopants, such as ZnO.

### 3.2. ZnO Incorporation on the Amorphous Carbon

**Figure 2:**
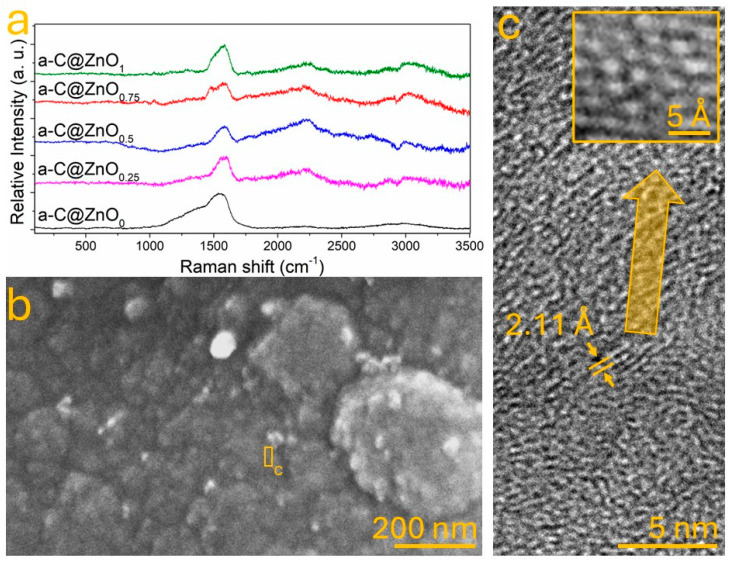
(**a**) Raman spectra comparison of a-C samples with varying ZnO content; the sample containing 0.75% ZnO is shown in (**b**) imaged by FESEM and (**c**) imaged by HRTEM. Note that the inset in (**c**) displays a HRTEM image with the interaction between Zn, O, and C atoms in the same sample.

Figure 2a presents the Raman spectra of a-C samples doped with varying ZnO contents referred to as weight (0%, 0.25%, 0.5%, 0.75%, and 1%). All spectra exhibit broad features characteristic of disordered carbon systems, with prominent D and G bands centered around ~1350 cm^−1^ and ~1580 cm^−1^, respectively. As ZnO content increases, the intensity of these bands becomes more pronounced, and the I_D_/I_G_ ratio increases progressively, suggesting enhanced defect density and a higher concentration of disordered sp^2^-hybridized domains. This trend is in line with previous studies, where metal oxide doping—especially with ZnO—has been reported to create strain and lattice distortions in carbon matrices, resulting in an increased number of active defect sites [34]. In particular, the sample doped with 0.75% ZnO shows a notable upshift and sharpening of the G band, which is often interpreted as evidence of improved π–π conjugation within the carbon framework, along with a modified local electronic structure due to Zn–O–C interactions [35]. This observation aligns with theoretical predictions by Mbonu et al., who reported that ZnO doping modulates the electronic band structure of carbon-based materials by introducing localized states favorable for charge separation and transport [36]. The FESEM image shown in Figure 2b corresponds to the 0.75% ZnO sample. The microstructure reveals a compact carbon matrix with granular features and distinguishable ZnO domains embedded within the amorphous carbon background. The morphological contrast between lighter ZnO-rich areas and the surrounding a-C phase suggests a heterogeneous distribution of the dopant, consistent with partial Zn diffusion and nucleation during the hydrothermal process [37]. Furthermore, Figure 2c provides HRTEM evidence supporting this interpretation. The high-resolution image reveals a continuous carbon network interspersed with nanometric crystalline regions exhibiting lattice fringes with a spacing of 2.11 Å, which corresponds to the (101) plane of the ZnO wurtzite structure, as reported in the JCPDS card no. 36-1451 [38]. The insert in Figure 2c further highlights the atomic-scale interaction between Zn, O, and C atoms. The periodic contrast in this region—where brighter spots correlate with Zn atoms and intermediate gray intensities with oxygen—suggests the formation of Zn–O–C bonding environments at the a-C interface. Such linkages have been theoretically and experimentally shown to play a pivotal role in facilitating interfacial charge transfer in metal oxide–carbon heterostructures [39].

### 3.3. Chemical Bonding Between ZnO and Amorphous Carbon

**Figure 3:**
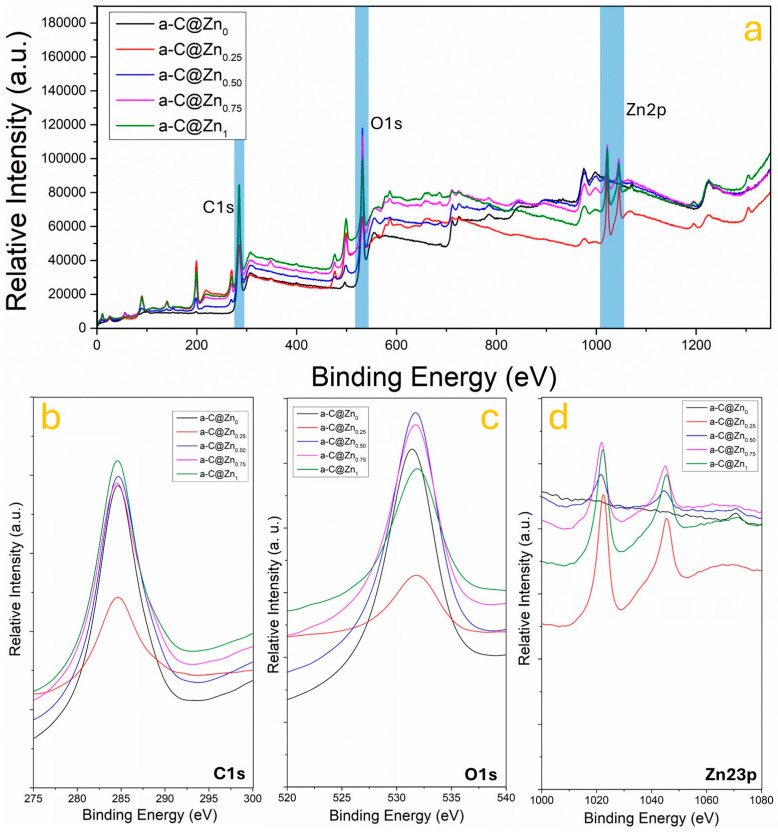
(**a**) XPS survey spectra of a-C@ZnO composites, (**b**) C 1s, (**c**) O 1s, and (**d**) Zn 2p binding energy bands range.

To investigate the chemical interaction between ZnO and the amorphous carbon (a-C) matrix, X-ray photoelectron spectroscopy (XPS) was employed. The survey and high-resolution spectra provided direct evidence of elemental composition, chemical states, and interfacial bonding evolution with increasing ZnO content. Figure 3a shows the full-range XPS spectra of the a-C@ZnO composites with ZnO concentrations of 0%, 0.25%, 0.50%, 0.75%, and 1.0%. Three prominent regions are identified: C 1s (~285 eV), O 1s (~532 eV), and Zn 2p (~1021 eV) [23,40]. A progressive increase in O 1s and Zn 2p intensities is observed with higher ZnO doping, confirming the successful incorporation of ZnO into the carbon matrix. Figure 3b presents the high-resolution C 1s spectra for all samples. The primary peak around 284.6 eV corresponds to C–C and C=C bonds (sp^2^- and sp^3^-hybridized carbon) [41,42], typical of amorphous carbon. With ZnO incorporation, a noticeable increase in the shoulder near ~286.1 eV is observed, attributed to C–O and C–OH groups, while a secondary peak at ~287.8 eV suggests the formation of C=O and O–C=O species [43]. These features indicate oxidation of the carbon surface, likely promoted by the presence of Zn^2+^ ions and oxygen species during hydrothermal synthesis. The progressive enhancement of signal related to oxygen besides the increasing ZnO content supports the formation of chemical linkages at the ZnO–a-C interface, particularly Zn–O–C bonds, as previously proposed for similar hybrid systems [23,44]. On the other hand, the O 1s spectra in Figure 3c provide further evidence of interfacial bonding and oxygen chemistry. All samples show a dominant peak centered around ~532.5 eV, attributed to oxygen in hydroxyl (–OH), C–O–C, and C=O environments [45]. For doped samples, the O 1s signal becomes sharper and more intense, especially at 0.75% ZnO content, indicating an increase in oxygen coordination likely due to the integration of ZnO particles. This enhancement is consistent with prior studies where O 1s components near 532 eV are associated with Zn–O bonding in hybrid nanostructures [46]. The absence of a distinct lattice oxygen peak at lower binding energy (~530 eV) further suggests that the ZnO domains are ultrasmall or amorphously dispersed, rather than existing as bulk crystalline ZnO. Figure 3d shows the Zn 2p spectra across the composite series. Two characteristic peaks at ~1021.5 eV (Zn 2p_3_/_2_) and ~1044.6 eV (Zn 2p_1_/_2_) are evident in all doped samples, with increasing intensity proportional to the ZnO content. These peaks confirm the presence of Zn^2+^ in a ZnO-like environment, consistent with literature values [47]. Notably, the absence of satellite peaks or binding energy shifts suggests that Zn remains chemically stable within the oxide state and does not form metallic Zn or hydroxide phases. The trend in intensity and peak symmetry supports the notion that Zn^2+^ species are well-integrated into the a-C matrix, potentially occupying interstitial positions or binding at defect-rich oxygenated sites [48].

The combined C 1s, O 1s, and Zn 2p XPS results provide clear evidence of chemical bonding between ZnO and the amorphous carbon matrix. As ZnO content increases, the emergence of oxygen-rich functional groups and the stabilization of Zn^2+^ in the ZnO form indicate strong interfacial coupling [49]. These findings are consistent with a Zn–O–C bond formation model, where ZnO nanodomains interact with defect sites and oxygen-containing groups on the carbon surface. Such interactions are crucial for facilitating interfacial charge transfer, a key mechanism for improving photocatalytic activity, as they enable efficient electron extraction from the carbon phase into the semiconductor domains. In particular, the a-C@ZnO_0.75_ sample shows the most significant intensity enhancements across all spectral regions, suggesting that this composition achieves the optimal balance between ZnO content and interfacial bonding.

### 3.4. Influence of ZnO on the Photocatalytic Activity

**Figure 4:**
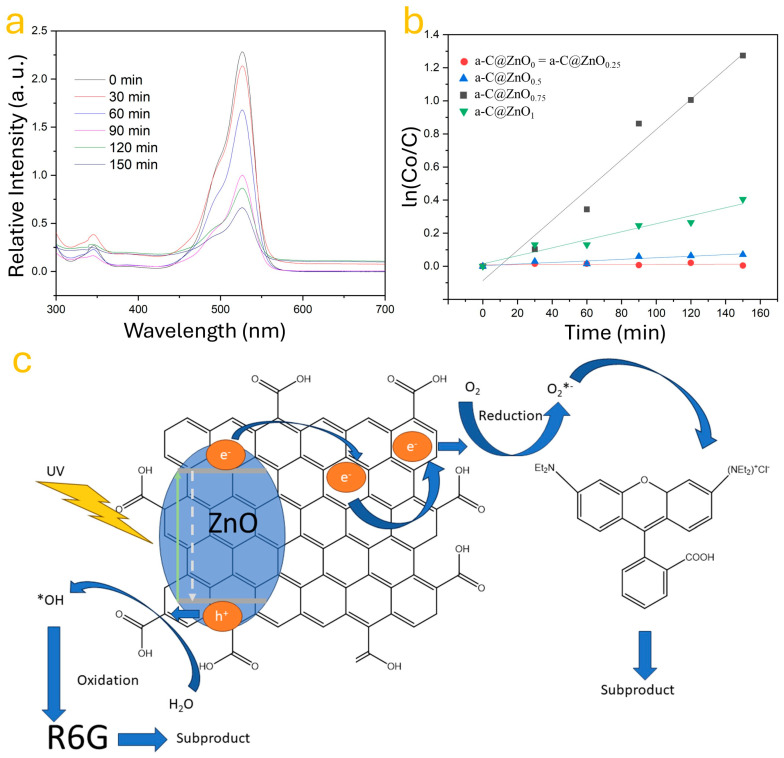
(**a**) The photocatalytic degradation of R6G by the a-C@ZnO_0.75_ sample, (**b**) a comparative analysis of the kinetic reactions in the photodegradation of R6G, and (**c**) the underlying mechanism of R6G’s photodegradation. Note that * point the reactive species generated.

The effect of ZnO incorporation on the photocatalytic activity of amorphous carbon (a-C) was evaluated through the degradation of rhodamine 6G (R6G) under UV irradiation. The experimental setup was designed to assess how varying ZnO concentrations influence the photodegradation efficiency and charge transfer behavior of the synthesized a-C@ZnO composites. Figure 4 summarizes the results through UV–Vis absorbance, reaction kinetics, and a mechanistic schematic of the photodegradation process. Figure 4a shows the evolution of the UV–Vis absorbance spectra of the R6G solution in the presence of the a-C@ZnO_0.75_ sample at different time intervals (0 to 150 min). A pronounced and continuous decrease in the absorption peak at approximately 528 nm, corresponding to the maximum absorbance of R6G, confirms the progressive photodegradation of the dye [50]. This degradation process is attributed to the catalytic activity of the a-C@ZnO hybrid under UV illumination, where the ZnO component facilitates electron–hole pair generation, and the a-C matrix contributes to charge separation and transport [51]. To quantify the photocatalytic performance, the degradation kinetics of R6G were analyzed using a pseudo-first-order model. Figure 4b plots the values of ln(C_0_/C) versus time for all studied samples. The undoped a-C sample (a-C@ZnO_0_) and the sample with 0.25 wt.% ZnO showed negligible photocatalytic activity, as indicated by the almost flat slope of the corresponding kinetic curves. In contrast, the photocatalytic activity increased significantly with ZnO content, reaching its peak at 0.75 wt.% ZnO (a-C@ZnO_0.75_), which showed the steepest linear fit and the highest degradation rate constant [52]. Beyond this concentration (i.e., at 1 wt.%), a decrease in the slope is observed, suggesting that excessive ZnO loading may lead to agglomeration or increased recombination centers, which can hinder effective charge transfer and reduce the availability of active sites [53]. The mechanistic pathway proposed for the degradation of R6G is illustrated in Figure 4c. Upon UV exposure, ZnO absorbs photons with energy equal to or greater than its bandgap (~3.37 eV), resulting in the generation of electron–hole pairs. In the composite structure, the photogenerated electrons (e^−^) are efficiently transferred to the a-C matrix due to its excellent electronic conductivity and a defect-rich structure, which helps prevent recombination. Simultaneously, the holes (h^+^) in ZnO can oxidize water molecules to produce hydroxyl radicals (•OH), while the transferred electrons reduce adsorbed oxygen molecules to form superoxide radicals (O_2_•^−^). These reactive oxygen species (ROSs) actively attack the R6G molecules, breaking down their chromophore structures into non-toxic subproducts. Importantly, the Zn–O–C interfacial bonds, previously identified by XPS and supported by HRTEM analysis, likely play a crucial role in facilitating interfacial electron transfer, stabilizing the charge carriers, and enabling prolonged radical generation. The hybrid architecture offers a synergistic platform where ZnO serves as the active photocatalyst and a-C functions as an electron mediator and adsorption scaffold. Overall, these results confirm that moderate ZnO doping (specifically at 0.75 wt.%) significantly enhances the photocatalytic degradation of R6G. This enhancement is attributed to the optimal dispersion of ZnO within the a-C matrix, improved charge separation dynamics, and the efficient formation of reactive radicals. The findings validate the potential of the a-C@ZnO_0.75_ composite as an effective, low-cost photocatalyst for environmental remediation, especially for the removal of organic dyes from wastewater. The results show an enhancement in the charge transfer based on the ZnO contents in the samples.

In addition, the incorporation of ZnO into the amorphous carbon (a-C) matrix plays a similar role to that of Fe–ZrO_2_ in the referenced system. The presence of ZnO in the a-C structure forms Zn–O–C interfacial bonds, which facilitates charge carrier separation by reducing electron–hole recombination, thus enhancing photocatalytic degradation efficiency. This mechanism is further supported by the improved rate constant observed in the a-C@ZnO_0.75_ sample [54]. On the other hand, one limitation of the present study is the absence of direct optical bandgap measurements for the ZnO-doped amorphous carbon (a-C@ZnO) composites. While the enhanced photocatalytic activity suggests improved charge transfer dynamics due to ZnO incorporation, a quantitative estimation of the optical bandgap is essential to establish a clearer understanding of the electronic transitions and photoexcitation behavior. In future work, we intend to employ UV–Vis diffuse reflectance spectroscopy (DRS) to determine the bandgap energies through Tauc plot analysis [55]. While the synthesized a-C@ZnO nanocomposites demonstrated promising photocatalytic activity under UV irradiation, an important environmental concern associated with the practical deployment of metal oxide-based photocatalysts is the potential leaching of metal ions—particularly Zn^2+^—into treated water. The release of zinc ions, even at trace levels, can pose ecotoxicological risks and compromise the sustainability of the photocatalytic process. This concern has been extensively studied in the context of g-C_3_N_4_-based heterostructures [56], where defect engineering and surface functionalization have been proposed to improve metal retention and photocatalyst stability. Analogously, the Zn–O–C bonding network identified in our ZnO-impurified amorphous carbon matrix may serve as a stabilizing interface, mitigating the migration of Zn^2+^ ions during photocatalysis. These covalent or partially ionic interactions can act as anchoring points, preventing the detachment of ZnO particles and thus limiting their solubilization under irradiation. Then, while numerous studies in the literature have focused on identifying the intermediate degradation products and complete mineralization pathways of organic dyes during photocatalysis, such as through total organic carbon (TOC) analysis or chromatographic techniques [57], the aim of the present work was not to conduct a comprehensive evaluation of the subproducts. Instead, the scope of this research was centered on evaluating photocatalytic activity as an indirect measure of charge transfer efficiency in ZnO-impurified amorphous carbon systems. By monitoring the degradation kinetics of rhodamine 6G (R6G) under UV light, we aimed to assess the influence of ZnO incorporation on electron–hole separation dynamics. Future work will be directed at elucidating the complete degradation mechanism, including the identification of by-products and assessment of mineralization, to provide a more in-depth understanding of the environmental implications of the photocatalytic process.

This study presents a novel, environmentally friendly one-pot hydrothermal approach for synthesizing ZnO-doped amorphous carbon (a-C@ZnO) composites, avoiding the need for aggressive oxidation or multi-step procedures commonly used in carbon-based photocatalysts. Unlike conventional composites based on reduced graphene oxide or graphitic carbon, this work emphasizes the use of an underexplored amorphous carbon matrix, offering greater biocompatibility and aqueous stability. A key innovation lies in the systematic investigation of ZnO loading effects—ranging from 0 to 1 wt.%—on the structural, chemical, and photocatalytic properties of the material. Through high-resolution transmission electron microscopy (HRTEM), Raman spectroscopy, and X-ray photoelectron spectroscopy (XPS), we elucidate the formation of Zn–O–C interfacial bonds, providing insight into the charge transfer mechanisms within the composite. The enhanced photocatalytic performance, particularly at 0.75 wt.% ZnO, is correlated with the structural and chemical evolution of the material, offering a new understanding of dopant–matrix interactions in non-crystalline systems. This work advances the design of cost-effective, low-crystallinity photocatalysts for environmental remediation and opens opportunities for future studies on heterostructures involving amorphous carbon phases. Future work will focus on assessing the long-term stability of the composites, exploring their activity under visible light, and optimizing the synthesis parameters to further enhance photocatalytic performance. In the context of this study, photocatalytic activity serves as an effective and indirect indicator for evaluating charge transfer behavior within ZnO-doped amorphous carbon (a-C@ZnO) systems. Under ultraviolet (UV) irradiation, the generation, separation, and migration of photogenerated electron–hole pairs are central to initiating redox reactions at the catalyst surface. The efficiency of these processes is intrinsically linked to the material’s charge transfer dynamics. Specifically, enhanced photocatalytic degradation of model pollutants, such as rhodamine 6G (R6G), reflects a reduced rate of electron–hole recombination and improved charge mobility across the ZnO–a-C interface. Given the challenges of directly probing charge transfer at the nanoscale, photocatalytic testing provides a practical and relevant method to assess the functional performance of the material. This approach is particularly suitable for the present work, as it integrates both structural features and chemical interactions—such as Zn–O–C bonding—that are hypothesized to influence electronic behavior. Thus, the observed variations in degradation efficiency across samples with different ZnO contents offer insight into the extent and nature of charge transfer enhancements induced by compositional tuning.

## 4. Conclusions

In this work, a novel one-pot hydrothermal strategy was successfully employed to synthesize ZnO-doped amorphous carbon (a-C@ZnO) composites with tunable zinc contents. Structural and chemical analyses confirmed the effective incorporation of ZnO into the a-C matrix, accompanied by the formation of Zn–O–C bonds that promoted interfacial charge transfer. SEM and Raman spectroscopy revealed morphological modifications and increased structural disorder due to ZnO doping, while XPS results indicated the presence of oxygen-rich functional groups that facilitate photocatalytic reactions. Photocatalytic evaluation through the degradation of rhodamine 6G under UV irradiation demonstrated a substantial improvement in activity for ZnO-doped samples compared to pristine a-C, with the a-C@ZnO_0.75_ composite achieving a maximum degradation efficiency of 72% and a significant increase in the kinetic rate constant. The enhanced performance is attributed to improved electron–hole separation and the generation of reactive oxygen species, promoted by the chemical interaction between ZnO and amorphous carbon. These findings underscore the potential of ZnO-doped amorphous carbon as an efficient, low-cost photocatalyst for environmental remediation applications.

## Data Availability

All data are available under request to the corresponding authors.
